# Radiomics Predicts for Distant Metastasis in Locally Advanced Human Papillomavirus-Positive Oropharyngeal Squamous Cell Carcinoma

**DOI:** 10.3390/cancers13225689

**Published:** 2021-11-14

**Authors:** Benjamin Rich, Jianfeng Huang, Yidong Yang, William Jin, Perry Johnson, Lora Wang, Fei Yang

**Affiliations:** 1 Department of Radiation Oncology, University of Miami, Miami, FL 33136, USA; benjamin.rich@jhsmiami.org (B.R.); william.jin@jhsmiami.org (W.J.); lora.wang@miami.edu (L.W.); 2 Department of Radiation Oncology, Affiliated Hospital of Jiangnan University, Wuxi 214125, China; Jianfengh2021@foxmail.com; 3 Department of Radiation Oncology, The First Affiliated Hospital of University of Science and Technology of China, Hefei 230026, China; ydyang@ustc.edu.cn; 4 Department of Radiation Oncology, University of Florida, Jacksonville, FL 32209, USA; perryjohnson@ufl.edu

**Keywords:** HPV, oropharyngeal cancer, radiomics, predictive model, chemoradiation, de-escalation

## Abstract

**Simple Summary:**

There is strong evidence that locally advanced human papillomavirus positive (HPV+) oropharyngeal squamous cell carcinoma (OPSCC) carries a significantly better prognosis than HPV negative OPSCC, suggesting the possibility of treatment de-escalation and, therefore, toxicity reduction in this patient population. The lack of success in clinical trials towards this end presses the need to risk stratify locally advanced HPV+ OPSCC patients who can safely have treatment de-escalated. The present study had recourse to radiomics for this purpose and showed that radiomics has the ability to discriminate patients with locally advanced HPV+ OPSCC who went on to develop distant metastasis after completion of definitive chemoradiation or radiation alone. The implications of this study aid in demonstrating the potential pivotal role of radiomics in predictive risk assessment and personalizing therapy for this patient population.

**Abstract:**

(1) Background and purpose: clinical trials have unsuccessfully tried to de-escalate treatment in locally advanced human papillomavirus positive (HPV+) oropharyngeal squamous cell carcinoma (OPSCC) with the goal of reducing treatment toxicity. The aim of this study was to explore the role of radiomics for risk stratification in this patient population to guide treatment. (2) Methods: the study population consisted of 225 patients with locally advanced HPV+ OPSCC treated with curative-intent radiation or chemoradiation therapy. Appearance of distant metastasis was used as the endpoint event. Radiomics data were extracted from the gross tumor volumes (GTVs) identified on the planning CT, with gray level being discretized using three different bin widths (8, 16, and 32). The data extracted for the groups with and without distant metastasis were subsequently balanced using three different algorithms including synthetic minority over-sampling technique (SMOTE), adaptive synthetic sampling (ADASYN), and borderline SMOTE. From these different combinations, a total of nine radiomics datasets were derived. Top features that minimized redundancy while maximizing relevance to the endpoint were selected individually and collectively for the nine radiomics datasets to build support vector machine (SVM) based predictive classifiers. Performance of the developed classifiers was evaluated by receiver operating characteristic (ROC) curve analysis. (3) Results: of the 225 locally advanced HPV+ OPSCC patients being studied, 9.3% had developed distant metastases at last follow-up. SVM classifiers built for the nine radiomics dataset using either their own respective top features or the top consensus ones were all able to differentiate the two cohorts at a level of excellence or beyond, with ROC area under curve (AUC) ranging from 0.84 to 0.95 (median = 0.90). ROC comparisons further revealed that the majority of the built classifiers did not distinguish the two cohorts significantly better than each other. (4) Conclusions: radiomics demonstrated discriminative ability in distinguishing patients with locally advanced HPV+ OPSCC who went on to develop distant metastasis after completion of definitive chemoradiation or radiation alone and may serve to risk stratify this patient population with the purpose of guiding the appropriate therapy.

## 1. Introduction

The incidence of oropharyngeal cancer has been increasing in the US due to the rising prevalence of human papillomavirus (HPV) [1]. Treatment for locally advanced oropharyngeal cancer consists of primary surgery or chemoradiation therapy with no randomized data demonstrating a survival difference between modalities; however, definitive chemoradiation is preferred due to less morbidity [2,3]. Patients with locally advanced oropharyngeal cancer who receive definitive radiation therapy have a range of outcomes with a 5-year overall survival of between 56 and 89% [4] and a rate of distant metastasis between 8 and 15% at 3 years [5]. HPV positive (HPV+) oropharyngeal squamous cell carcinoma (OPSCC) has a significantly better prognosis compared to their HPV-negative counterparts [5]. Although additional clinical factors, such as stage, performance status, smoking history, and age, have also been shown to be independently associated with cancer-related outcomes in oropharyngeal cancer [6], other high-risk pathological factors may not be prognostic in HPV+ OPSCC [7]. Finding HPV+ OPSCC patients with a favorable prognosis may allow for de-escalation of treatment and, thereby, reduce treatment-related morbidity. Indeed, this is the goal of a number of current and recently completed HPV+ OPSCC clinical trials [8,9,10].

Imaging with magnetic resonance imaging (MRI) or computed tomography (CT) of the head and neck (HN) is becoming the standard of care in the diagnosis and staging of HPV+ OPSCC, with 2-deoxy-2-[18F] fluoro-D-glucose (FDG) positron emission tomography (PET) scan reserved for the evaluation of nodal or distant metastasis [11]. To better harness the potential of this rich and abundant radiological data of these patients, radiomics has been deployed in an attempt to address a series of longstanding challenges in the treatment and management of oropharyngeal cancer [12,13,14]. Radiomics involves extracting quantitative imaging features from radiological data to aggregate into databases and, after validation, rendering sophisticated clinical information [15,16]. It was reported that radiomics from pre-treatment PET scans may be superior to clinical staging variables at predicting progression and overall survival in oropharyngeal cancer patients [12]. Radiomics data from PET scans combined with clinical data were found to be associated with predictive value for HN cancer locoregional recurrence, while conventional CT-based radiomics data might be predictive of distant recurrence [17]. Furthermore, radiomic models utilizing CT images evaluating both the primary HN tumor, as well as metastatic disease in regional lymph nodes, were observed with improved predictive power for locoregional control relative to models assessing the primary tumor alone [18].

Distant metastasis is a clinically relevant outcome because salvage therapies are usually limited to systemic therapy with poor outcomes [19]. However, the most common site of first treatment failure is locoregional and, as mentioned previously, less than 10% of patients with locally advanced HPV+ OPSCC will develop a distant metastasis at three years [5]. As a result, the majority of patients with HPV+ OPSCC may be overtreated. With cancer care entering into an era of precision medicine enabling tailored effective treatments, interventions, and models for prevention [20,21,22], there is a need to identify locally advanced HPV+ OPSCC patients for whom treatment may be safely de-escalated. In view of this, the current study aimed to explore and evaluate the prognostic value of radiomics parameters derived from baseline CT scans for the prediction of distant metastasis in patients with HPV+ OPSCC treated with definitive radiation or chemoradiation therapy.

## 2. Materials and Methods

### 2.1. Patient Population and Selection

The current study employed patient data accrued within the HNSCC collection for which imaging and clinical data are publicly available from The Cancer Imaging Archive (TCIA). Ethical approval for this study was deemed unnecessary by the local institutional review board (IRB), on account of only publicly available aggregated patient data being utilized. The dataset comprised of planning fan beam CT (FBCT) of 627 OPSCC patients undergoing concurrent chemoradiation or radiation alone with curative intent at the MD Anderson Cancer Center, from 2005 through 2012 [23,24]. Patients who met any of the following criteria were excluded from the analysis: (1) unknown distant metastasis status; (2) HPV status unknown or negative; (3) presence of CT artifacts, i.e., streak artifacts due to dental fillings, among others, within the primary tumor volume, (4) primary tumor volume less than 0.5 cm^3^; and (5) early-stage disease (Stage I and II) according to American Joint Committee on Cancer (AJCC), 7th edition [25]. It should be noted that in the present study the clinical stage was not updated to the AJCC 8th edition in view of the fact that the AJCC 7th edition staging currently still guides definitive chemoradiation therapy recommendations for locally advanced HPV-related oropharyngeal cancer [11]. Applying these criteria, the final cohort consisted of a set of 225 patients out of the original 627 patients. FBCT images for radiotherapy planning of the selected patients were acquired from nine different scanners, from four different manufacturers (General Electric Co., Milwaukee, WI, USA; Philips Healthcare, Best, The Netherlands; Siemens Medical System, Erlangen, Germany; and Toshiba Medical System, Otawara, Japan), with exposure of modulated mAs in the range of 139–350 at 120 kVp. The included CT scans featured a slice thickness of 0.5–3.75 mm and in-plane spacing from 0.41 mm to 0.98 mm. For a detailed description of the imaging protocols being used for this collection of data please refer to Grossberg et al. [26]. Clinical characteristics of the included patient population are summarized in Table 1.

### 2.2. Radiomics Feature Extraction

FBCT scans of the study cohort came along with primary gross tumor volume (GTV) already delineated by expert radiation oncologists. To repurpose the accompanying contouring data for radiomics analysis as was of interest of the present study, the primary GTV contours were further re-examined and revised, if necessary, according to the most updated target delineation guidelines [27], by a radiation oncology physician equipped with extensive clinical experience in contouring HN lesions as part of radiotherapy treatment planning practice. Note that only GTV was considered for feature extraction, given the inconsistence in lymph node involvement of the study population. Prior to the radiomics features’ extraction, images, and their corresponding primary GTV contours, were resampled to an isotropic voxel size of 1 mm. The fixed bin width method was adopted as the gray level discretization algorithm, as was recommended by others [28]. Given that there is still debate on the exact size of bin width that would be best suited for CT-based radiomics analysis, the present study took into account three sizes of bin width: 8, 16, and 32. The radiomics parameters being examined consisted of an array of volumetric texture features related to the gray level co-occurrence matrix (GLCM) with a voxel displacement of 1, gray level dependence matrix (GLDM) with a threshold for dependence of 0 and distance to neighbor of 1, gray-level run-length matrix (GLRLM) without distance weighting, and neighborhood gray-tone difference matrix (NGTDM) with a neighborhood size of 3 × 3 × 3 [29,30,31,32,33,34,35,36,37]. GLCM-based texture features capture spatial properties of the image content at a local level with emphasis on the frequency of pairwise voxel interactions with respect to intensity value and spatial disposition. GLDM-based texture features quantify gray level spatial dependences, defined as the number of immediate neighboring voxels that have the same gray level with respect to the center voxel, within an image region of interest (ROI). GLRLM-based texture features depict spatial properties of the image content at a regional level with consideration of the spatial frequency of the contiguous voxels of identical gray-level values along different orientations. NGTDM-based features exploit the peculiarity of visual perception to detect spatial details of the image content in terms of gray level differences between an individual voxel and its local neighbors. After being extracted, the raw data of each radiomics feature further underwent standardized score (z-scores) normalization to mitigate, if not completely eliminate, the scale difference effects across features [38]. For a complete list of the radiomics features being employed please refer to Table 2.

### 2.3. Data Balancing and Feature Selection

Given that the two cohort groups were unbalanced with patients free from distant metastasis greatly outnumbering those who developed distant metastasis, the minority class was, thus, augmented to match up with the majority class in the number of the patients using three alternative approaches: synthetic minority oversampling technique (SMOTE), adaptive synthetic sampling (ADASYN), and borderline SMOTE. SMOTE generates synthetic minority class instances along the hyperline segments in the feature space that connect randomly picked instances and their preset number of nearest neighbors [39]. ADASYN adaptively generates synthetic instances based on the density of the minority class in the feature space such that more synthetic instances are generated in the region of the feature space where the density of minority class instances is low and fewer where the density is high [40]. Borderline SMOTE employs a similar strategy while operating only on so-called “danger” instances, i.e., the ones at least half of whose nearest neighbors are from the minority class, resulting in more instances being synthesized in the vicinity of the boundary between the two classes than in the interior of the minority class [41]. With three different gray level discretization schemes and three different data balancing algorithms, a total of nine radiomics datasets were established, with each comprising 59 distinct radiomics features. As for feature selection, the aim was to form, from all the radiomics features being extracted, a relatively small subset of features that are capable of retaining the overall discriminatory power of all original features. To achieve this, the minimum redundancy maximum relevance (mRMR) feature selection algorithm [42] was adopted. It minimizes the redundancy of the feature set while maximizing the relevance to the response variable, which in this case, was the distant metastasis status. Feature selection was carried out for each individual radiomics dataset with the top five ranked by mRMR with high relevance and low redundancy being retained and, also, collectively by identifying the top common five for radiomics datasets with a shared data balancing algorithm while differing in gray level discretization scheme.

### 2.4. Classifier Construction and Validation

**Figure 1:**
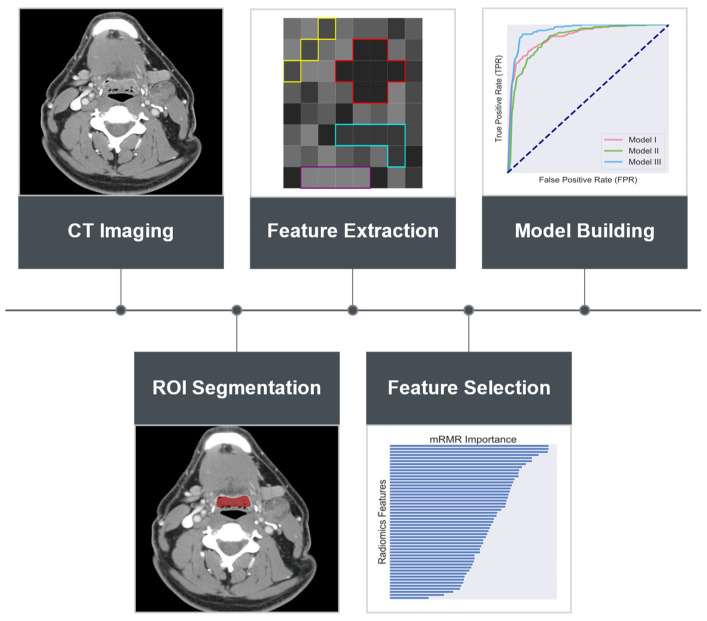
Workflow of the key steps in radiomics analysis using simulation CT for the differentiation of distant metastasis status in locally advanced human papillomavirus-positive (HPV+) oropharyngeal squamous cell carcinoma (OPSCC). Primary gross tumor volumes (GTVs) were delineated on the simulation CT for each of the studied patients. Radiomics features were then extracted from the identified primary GTV volumes. Feature selection was conducted employing minimum redundancy maximum relevance (mRMR) that prioritizes radiomics features with high relevance to the target class while low redundancy to each other, followed by building support vector machine (SVM)-based classifiers using the selected features.

Support vector machines (SVMs) with a Gaussian kernel were employed in the reduced feature space as the classifiers of choice to discriminate the two cohort groups. The rationale for adopting SVMs as the classifiers was primarily due to their ability in generating classification hyperplanes such that the margins between the hyperplane and the nearest instances of the classified sample categories are maximized. In doing so, it allows for achieving global optimal solutions and, hence, aids in the generalizations of the resultant classifiers. Moreover, SVMs are more robust to data biases and disturbances as well as in dealing with small sample sizes when compared with other machine learning classification techniques [43,44]. To prevent from overfitting, cross-validation (5-fold), repeated 10 times, was used to assess and validate the classification performance [45]. An overview of the key steps of the methods being used is summarized in Figure 1.

### 2.5. Statistical Analysis

Frequency counts and percentages were used to summarize categorical demographic characteristic variables of the studied patient population while medians and ranges were used for continuous variables. The selected radiomics features were compared between the two patient cohorts and examined for differences using the Kruskal–Wallis test, with the null hypothesis being there is an equal median in feature values between the two cohorts. Receiver operating characteristic curves (ROCs) were constructed as plots of the percentage of true-positives (sensitivity) against the percentage of false-positives (100-specificity), for each of the built classifiers. The discriminative ability of the developed classifiers was measured by the area under the ROC curve (AUC) with a value of 1 being perfect, greater than 0.9 being outstanding, between 0.8 and 0.9 being excellent, and between 0.7 and 0.8 being acceptable [46]. Discriminative ability was also compared between classifiers according to the methods for comparing the areas under correlated ROC as suggested by DeLong et al. [47] All statistical tests were two-sided, and *p* values of 0.05 or less, after multiple test correction using the method of Bonferroni–Holm, were considered as statistically significant [48]. All statistical analysis was performed using JMP Pro^®^ Version 12 statistical software package (SAS Institute Inc., Cary, NC, USA).

## 3. Results

A total of 225 OPSCC patients positive for HPV met the inclusion criteria and were included in the analysis. The majority of the patients were men (87%), with a median age at diagnosis of 57 years. The primary site of disease was the base of tongue in 118 patients, tonsil in 84, glossopharyngeal sulcus in 8, soft palate in 2, and 13 were not specified. In the study population, 39 (17%) patients had AJCC 7th edition stage III disease while 186 (83%) had non-metastatic stage IV disease. As to tumor (T) and nodal (N) statuses, the most frequently occurring were T2 (41%) and N2 (79%), respectively. With respect to the treatment regimen, 122 (54%) patients underwent concurrent chemoradiotherapy (CRT), 59 (26%) received induction chemotherapy followed by CRT, 30 (14%) had radiation alone, and the remaining 14 (6%) were treated with induction chemotherapy followed by radiation alone. For a detailed description of patient demographic and clinical characteristics please refer to Table 1. The median follow-up was 73.9 months. The median time to distant metastasis onset was 21.3 months (range: 8.2–136), and the median follow-up time for patients free from distant metastasis was 75.8 months (range: 6–143.2). At last follow-up, of the 225 patients included, 21 (9.3%) had developed distant metastases while the rest remained free from distant metastasis. Of the 21 patients that developed distant metastases, there were 19 males and 2 females, with a median age at diagnosis of 60 years. The primary site of disease of these 21 patients was as follows: base of tongue (13), tonsil (6), soft palate (1), and not specified (1). Regarding AJCC 7th edition staging, 2 out of the 21 patients presented with stage III disease while the remaining 19 had stage IV.

**Figure 2:**
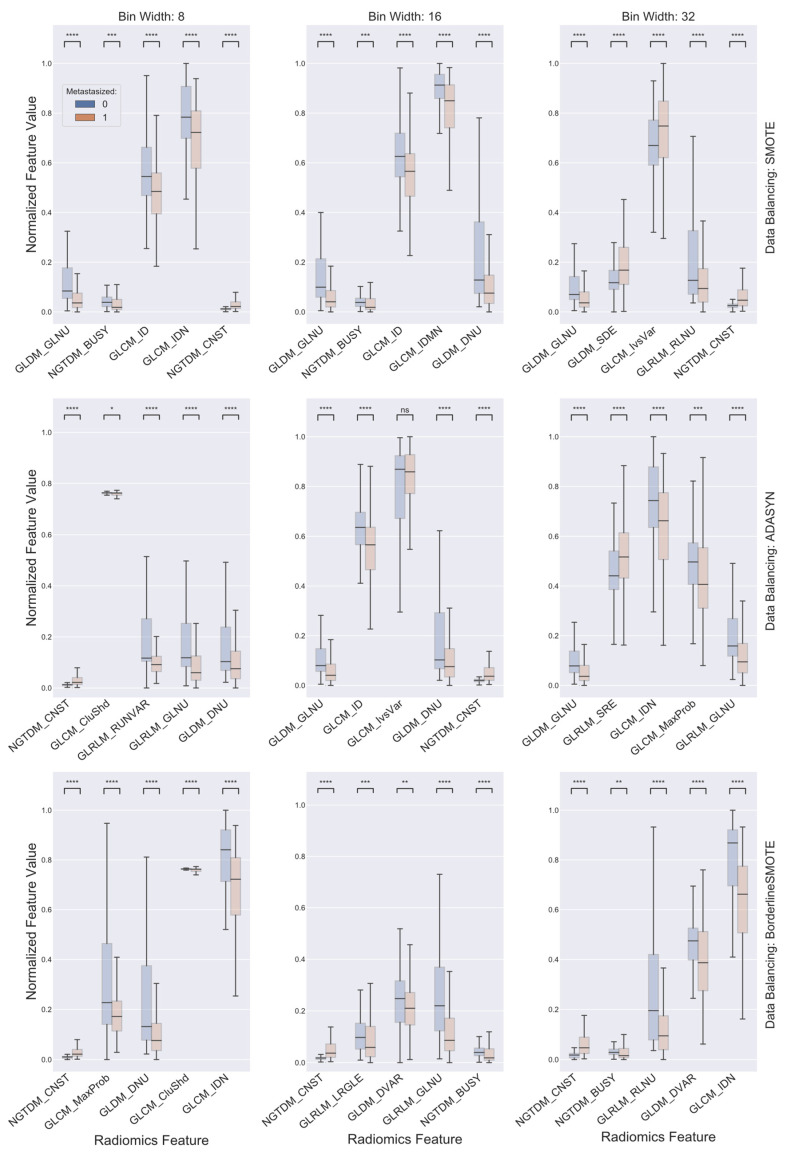
Boxplots comparing the top five radiomics features selected for each combination of gray level discretization schemes and data balancing algorithms between cohorts with (in red) and without (in blue) distant metastasis. The radiomics feature values were normalized to between 0 and 1 for visual display. On each box, the central mark indicates the median, and the top and bottom edges of the box indicate the 25th and 75th percentiles, respectively. For each box pair comparison: NS, not significant at level of 0.005; *, *p* < 0.005; **, *p* < 0.001; ***, *p* < 0.0001; and ****, *p* < 0.00001. All *p* values were corrected for multiple testing by the Bonferroni–Holm method.

The top five ranked features for each combination of gray level discretization schemes and data balancing algorithms are presented in Figure 2, with values being compared between the cohort classes with and without distant metastasis. Of note, first, the top discriminative features selected for a given combination of gray level discretization scheme and data balancing algorithm may span across multiple—or even all—feature categories being investigated. For instance, the selected top five features for the combination that had ADASYN as the data balancing algorithm and used a gray level discretization scheme with a bin width of 8 emerged from all four studied feature categories comprising of NGTDM-based contrast (CNST), GLRLM-based gray level nonuniformity (GLNU) and run variance (RUNVAR), GLCM-based cluster shade (CluShd), and GLDM-based dependence nonuniformity (DNU), indicating no single feature category has a dominant position in terms of differentiating the two cohorts. Secondly, note that almost all the selected radiomics features, except GLCM-based inverse variance (IvsVar) for the combination using ADASYN as the data balancing algorithm and a bin width of 16 for gray level discretization, differed significantly between the two patient cohorts according to the Kruskal–Wallis test with Bonferroni–Holm post hoc performed to correct for the number of comparisons. Furthermore, although the selected top discriminative features varied from one combination of gray level discretization scheme and data balancing algorithm to another, it is readily observable from Figure 2 that there existed certain shared common radiomics features among the top ones selected for different combinations of gray level discretization scheme and data balancing algorithm. Such as, for example, GLDM-based GLNU and NGTDM-based CNST emerged as one of the top features selected for five and seven of the nine distinct combinations, respectively.

**Figure 3:**
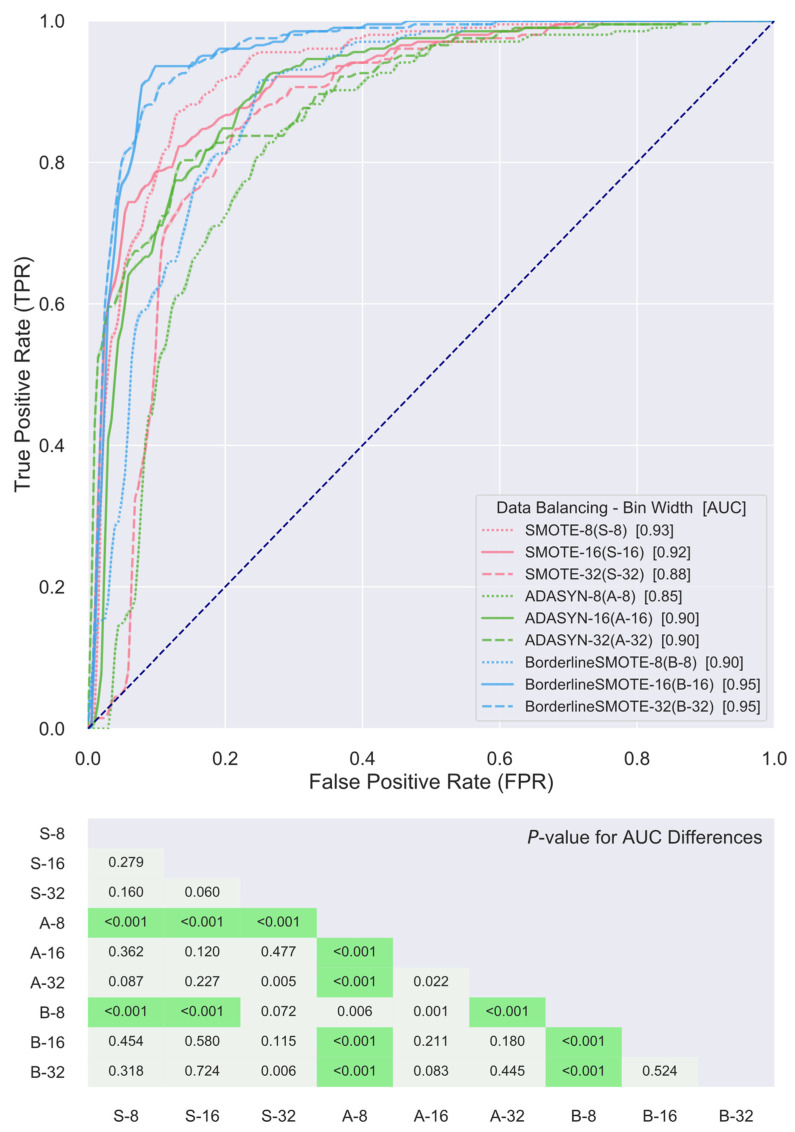
Receiver-operating characteristic (ROC) curve comparisons between support vector machine (SVM) classifiers utilizing the top five radiomics features selected for each combination of gray level discretization schemes and data balancing algorithms in differentiating cohorts with and without distant metastasis. The diagonal dash line from the bottom left to the top right corners represents the random classifier, to which the closer an ROC curve approximates the less powerful in differentiating the two cohorts is the corresponding classifier.

ROC curves of the SVM classifiers constructed for the nine distinct combinations of gray level discretization schemes and data balancing algorithms using their own respective top five radiomics features for the differentiation of patient cohorts with and without distant metastasis are presented in Figure 3. It is noted, first of all, that the AUC values of the ROC curves of all combinations are greater than or equal to 0.85, indicating that the obtained classifiers all achieved at least an excellent level in discriminating the two patient cohorts and, thus, evidencing that the underlying radiomics features selected are associated with predictive power for distant metastasis status in patients with locally advanced HPV+ OPSCC. Furthermore, comparisons of the AUCs by the DeLong test revealed that the resulting classifiers did not differentiate the two cohorts significantly better than each other with just a few exemptions. The AUC based on the combination using ADASYN as the data balancing algorithm and a bin width of eight for gray level discretization was significantly outperformed by the rest of the obtained classifiers. The combination using Borderline SMOTE as the data balancing algorithm and a bin width of eight for gray level discretization also showed significantly lower discriminative power in comparison to the rest, except for the ones based on the combination using SMOTE for data balancing and a bin width of 32 for gray level discretization and the combination using ADASYN for data balancing algorithm and a bin width of eight for gray level discretization.

**Figure 4:**
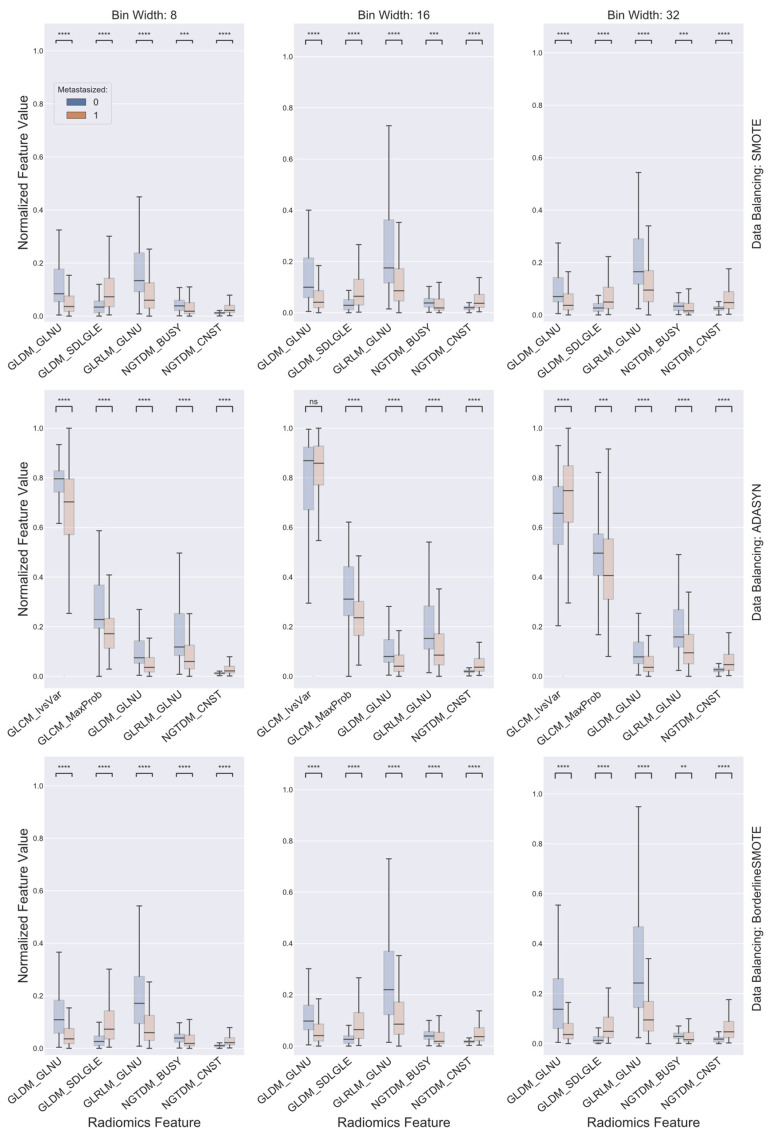
Boxplots comparing the top five consensus radiomics features selected for each of the data balancing algorithms between cohorts with (in red) and without (in blue) distant metastasis. The radiomics feature values were normalized to between 0 and 1 for easier visual display. On each box, the central mark indicates the median, and the top and bottom edges of the box indicate the 25th and 75th percentiles, respectively. For each box pair comparison: NS, not significant at level of 0.005; **, *p* < 0.001; ***, *p* < 0.0001; and ****, *p* < 0.00001. All *p* values were corrected for multiple testing by the Bonferroni-Holm method.

**Figure 5:**
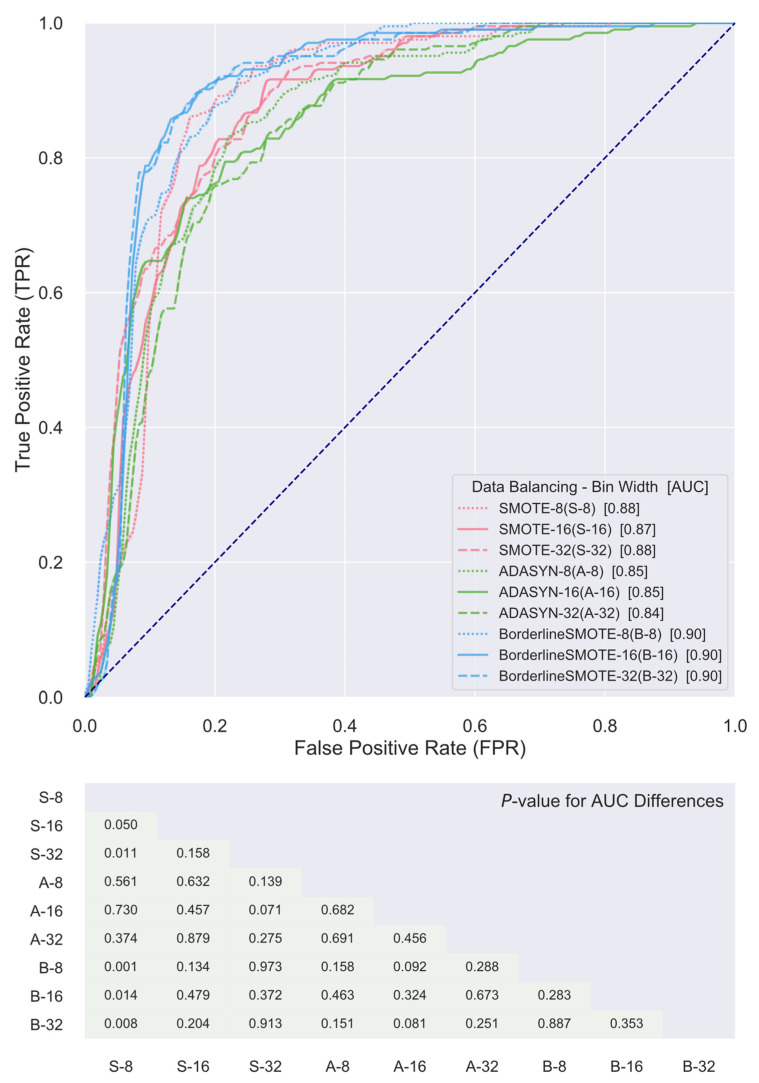
Receiver-operating characteristic (ROC) curve comparisons between support vector machine (SVM) classifiers utilizing the top five consensus radiomics features for each of the data balancing algorithms in differentiating cohorts with and without distant metastasis. The diagonal dash line from the bottom left to the top right corners represents the random classifier, to which the closer an ROC curve approximates, the less powerful in differentiating the two cohorts is the corresponding classifier.

The top five consensus features for combinations with a shared data balancing algorithm, while differing in gray level discretization scheme, are presented and compared between the two cohorts in Figure 4. First, it was evident that all the identified consensus features, except for GLCM-based IvsVar for the combination using ADASYN for the data balancing algorithm and a bin width of 16 for gray level discretization, demonstrated significant differences between the two patient cohorts, indicating the discriminative power for predicting distant metastasis failure of each individual consensus feature. Further, it is of note that the top five consensus features for combinations using SMOTE and Borderline SMOTE as the data balancing algorithm were identical and overlapped for three features, including GLDM-based GLNU, GLRLM-based GLNU, and NGTDM-based CNST, of the top five consensus features for combinations using ADASYN as the data balancing algorithm. ROC curves of the SVM classifiers established for the nine distinct combinations of gray level discretization scheme and data balancing algorithm using their corresponding top five consensus radiomics features for the differentiation of the two cohorts are presented in Figure 5. It can be observed that the AUC value of the ROC curves for all the combinations, though slightly poorer in comparison to their respective counterparts derived from SVM classifiers formed on their very own top five radiomics features as presented in Figure 3, were no less than 0.84 and, thus, attained a level of excellence in discriminating the two patient cohorts as well. In addition, comparisons of the AUCs by the DeLong test further showed that these classifiers did not show significant differences between each other in terms of differentiating the two cohorts of interest.

## 4. Discussion

The majority of oropharyngeal cancer patients treated with definitive radiation therapy report a high quality of life 5 years out from treatment [49]. However, long-term survivors continue to experience treatment-related toxicity, such as feeding tube dependence, xerostomia, and fibrosis [49,50,51]. Radiation treatment toxicities may occur many years after completing treatment. For example, osteoradionecrosis is experienced at a median of 7 years after completing radiation therapy [50]. Toxicities such as chronic pharyngeal and laryngeal dysfunction and feeding tube dependence are directly related with radiation dose to the hypopharynx and the total prescribed radiation dose [50,52]. Dose escalation with concurrent chemotherapy also contributes to treatment toxicities, leading to sequalae such as chronic opioid use [53,54]. Therefore, research is exploring avenues for chemoradiation treatment de-escalation to reduce treatment-related toxicities in the management of oropharyngeal cancer without compromising survival outcomes [55]. For example, in locally advanced HPV-related oropharyngeal cancer, a number of clinical trials have been motivated to de-escalate treatment with the aim of reducing treatment toxicities in the disease [56]. However, these trials have, so far, been unsuccessful in demonstrating a de-escalation treatment regimen in HPV+ OPSCC with equivalent clinical outcomes [9,10]. It may, therefore, be necessary to define a subset of HPV+ OPSCC patients who can safely have treatment de-escalated. This has been currently an active area of research [8,9,10,55]. Reported poor prognostic indicators for HPV+ OPSCC include higher anatomical stage, nodal disease, and extranodal extension among others [6,57,58]. In addition, tumor biomarkers such as expression of NOTCH1, tumor hypoxia, and genetic variants in genes such as TGFβ1, COX7A1, and TBX5 have also been shown to be associated with prognosis in HPV-related HN cancer [59,60,61,62]. Despite the prognostic associations of these factors, genomic testing is costly, invasive, oftentimes not widely available, and, moreover, comes along with an unproven ability to dictate treatment in HPV+ OPSCC [63].

The current study resorted to radiomics for this purpose. Radiomics has demonstrated utility in a wide range of oncologic applications such as risk stratification of cholangiocarcinoma preoperative lymph node metastasis, prediction of mortality in non-small cell lung cancer, assessment of response to chemoradiation in cervical cancer, and quantitative characterization of invasiveness of breast malignancies, among a great many others [64,65,66,67,68]. To investigate the ability of radiomics in the stratification of HPV+ OPSCC patients for treatment de-escalation, the present study adopted the appearance of distant metastasis as the endpoint event and assessed the prognostic value held, if any, by pretreatment CT-based radiomics features within a cohort population consisting of a total of 225 locally advanced HPV+ OPSCC patients treated with curative-intent radiation or chemoradiation. Despite considerable variability existing within the studied patient data regarding some of the key aspects of radiomics analysis, such as scanner vendor, acquisition protocol, and ROI contouring, among others, a number of consistent findings were seen to emerge. First, as previously mentioned, certain radiomics features, such as GLDM-based GLNU and NGTDM-based CNST, persistently appeared among the top selected features for a majority of the various combinations of gray level discretization scheme and data balancing algorithm. GLDM-based GLNU measures the similarity of gray level values within an ROI through quantifying gray level dependencies. As is readily seen in Figure 2, GLDM-based GLNU, if selected, exhibited a significantly larger value in the cohort that did not develop distant metastasis than was in the cohort with distant metastasis, indicting a more heterogeneous gray level dependency of the latter. As to NGTDM-based CNST, it is built on the quantification of the difference between a gray level value and the average gray level value of its neighbors and serves as a measure of the spatial gray level alternations within the ROI. NGTDM-based CNST, when selected, demonstrated significantly higher values in the cohort that developed distant metastasis in comparison with the cohort without metastasis, illustrating that the former is associated with much greater spatial change rate in gray levels and turns up more heterogeneity in terms of this measure. Furthermore, it was found that there existed common radiomics features among the top consensus ones being selected for combinations with a shared data balancing algorithm while differing in gray level discretization scheme. Besides the two radiomics features as alluded to above, GLRLM-based GLNU stood out as another one of these common shared features. As is shown in Figure 4, all three features were capable of distinguishing the two cohorts with statistical significance irrespective of gray level discretization scheme and data balancing algorithm being used, implying the discriminative and potentially predictive values of the underlying radiographic patterns being abstracted by these features. Most importantly, it was found that SVM classifiers built for the various combinations of discretization scheme and data balancing algorithm, using either the top consensus radiomics features or their own respective top ones, were all able to attain levels of excellence and beyond in differentiating the two cohorts. The ROC AUC of these classifiers ranged from 0.84 to 0.95, with a median of 0.90, as is illustrated in Figure 3 and Figure 5. These results compare favorably with other studies in predicting distant metastases in HN cancer [14,17,69].

All that being said, the current study shows that radiomics can play a role in determining distant metastasis failure in patients with locally advanced HPV+ OPSCC treated with curative-intent radiation therapy or chemoradiation therapy. In particular, as indicated earlier, radiomics features including GLDM-based GLNU, GLRLM-based GLNU, and NGTDM-based CNST emerged and stood out with significant discriminative power for distinguishing the two patient cohorts, almost regardless of what data balancing algorithm and gray level discretization schemes were combined and used. Features, as such, may serve as potentially useful adjuncts to conventional clinical risk factors in identifying patients and subgroups in the HPV+ OPSCC population at increased risk for distant metastasis. Possible applications of radiomics include guiding total radiation dose, elective radiation volumes, and receipt of chemotherapy. For example, a patient with an HPV+ OPSCC of the tongue base and low risk of distant metastases predicted by radiomics may be able to safely limit elective nodal radiation volumes on the contralateral side. Patients with comorbidities and competing non-malignancy causes of death may also benefit from treatment de-escalation [70,71]. Radiomics could help clinicians determine the appropriate patients with poor performance status who should receive treatment de-escalation. Ultimately, radiomics should become another tool in delivering precision medicine, where the treatment delivered is congruent with well-understood risks and benefits [20,21].

Strengths of the present study include a relatively large homogeneous population of patients with HPV+ OPSCC, all treated with definitive chemoradiation therapy or radiation therapy, and the consistency in cohort differentiation results. On the other hand, there are several limitations that should be considered when interpreting the findings. First, the presented analysis was conducted retrospectively on one set of patients from a single institution. As such, confounders inherent to the observational nature of the study design were unaccounted for and, hence, the use of the proposed predictive models for the prediction of distant metastasis status in HPV+ OPSCC needs to be prospectively confirmed in, ideally, a multicenter clinical trial setting. Furthermore, there exists a wide array of uncertainties and variabilities involved in radiomics analysis, ranging from the process of image acquisition to tumor delineation as well as feature extraction. The impact due to stochastic fluctuations of these processes on the proposed predictive models was not explicitly assessed in the present study; however, the robustness of the proposed models appeared to be reasonable given that (1) the underlying imaging data were acquired on multiple scanners from different vendors using various heterogeneous imaging protocols; (2) the tumor contour data used by the study for radiomics parameter extraction were provided by diverse expert radiation oncologists over a time span of approximately a half decade; and (3) there existed a consistency in the radiomics features selected as being the most predictive for various different combinations of gray level discretization scheme and data balancing algorithm. Finally, the current study included only patients being treated with definitive chemoradiation or radiation alone, so the findings may be more applicable, if not entirely limited, to intact HPV+ OPSCC.

## 5. Conclusions

The demonstrated discriminative ability of radiomics in distinguishing patients with locally advanced HPV+ OPSCC who went on to develop distant metastasis after completion of definitive chemoradiation or radiation alone indicates the potential of radiomics in predictive risk assessment for distant failure. Radiomics may provide another avenue in stratifying this patient population for treatment de-escalation.

## Figures and Tables

**Table 1:** Patient demographic and clinical characteristics (n = 225).

Demographic		Median or Occurrence	Range or Percentage
Sex	Male	195	87%
	Female	30	13%
Age at diagnosis (y)		57	38–81
Smoking history	Never	88	40%
	Former	82	36%
	Current	55	24%
Tumor subsite	Base of tongue	118	52%
	Tonsil	84	37%
	Not specified	13	6%
	Glossopharyngeal sulcus	8	4%
	Soft palate	2	1%
AJCC stage	III	39	17%
	IV	186	83%
T category	T1	56	25%
	T2	93	41%
	T3	44	20%
	T4	32	14%
N category	N0	11	5%
	N1	32	14%
	N2	178	79%
	N3	4	2%
Treatment regimen	Radiation alone	30	14%
	Concurrent chemoradiotherapy (CRT)	122	54%
	Induction chemotherapy and radiation alone	14	6%
	Induction chemotherapy and concurrent CRT	59	26%

**Table 2:** Radiomics features being studied.

Category	Feature
Gray-level Co-occurrence Matrix (GLCM)	Autocorrelation (AutoCorr), Cluster Prominence (CluProm), Cluster Shade (CluShd), Cluster Tendency (CluTndy), Contrast (CNST), Correlation (CORR), Difference Average (DiffAvg), Difference Entropy (DiffEpy), Difference Variance (DiffVar), Inverse Difference (ID), Inverse Difference Moment (IDM), Inverse Difference Moment Normalized (IDMN), Inverse Difference Normalized (IDN), Informational Measure of Correlation (IMC1), Informational Measure of Correlation (IMC2), Inverse Variance (IvsVar), Joint Average (JntAvg), Joint Energy (JntEngy), Joint Entropy (JntEpy), Maximal Correlation Coefficient (MCC), Maximum Probability (MaxProb), Sum Average (SumAvg), Sum Entropy (SumEpy), Sum Squares (SumSqr)
Gray-level Dependence Matrix (GLDM)	Dependence Entropy (DEPNEPY), Dependence Nonuniformity (DNU), Dependence Nonuniformity Normalized (DNUN), Dependence Variance (DVAR), Gray Level Nonuniformity (GLNU), Gray Level Variance (GLV), High Gray Level Emphasis (HGLE), Large Dependence Emphasis (LDE), Large Dependence High Gray Level Emphasis (LDHGLE), Large Dependence Low Gray Level Emphasis (LDLGLE), Low Gray Level Emphasis (LGLE), Small Dependence Emphasis (SDE), Small Dependence High Gray Level Emphasis (SDHGLE), Small Dependence Low Gray Level Emphasis (SDLGLE)
Gray-level Run Length Matrix (GLRLM)	Gray Level Nonuniformity (GLNU), Gray Level Nonuniformity Normalized (GLNUN), Gray Level Variance (GLV), High Gray Level Run Emphasis (HGLRE), Long Run Emphasis (LRE), Long Run High Gray Level Emphasis (LRHGLE), Long Run Low Gray Level Emphasis (LRLGLE), Low Gray Level Run Emphasis (LGLRE), Run Entropy (REPY), Run Length Nonuniformity (RLNU), Run Length Nonuniformity Normalized (RLNUN), Run Percentage (RP), Run Variance (RUNVAR), Short Run Emphasis (SRE), Short Run High Gray Level Emphasis (SRHGLE), Short Run Low Gray Level Emphasis (SRLGLE)
Neighboring Gray Tone Difference Matrix (NGTDM)	Busyness (BUSY), Coarseness (COAS), Complexity (CPLX), Contrast (CNST), Strength (STR)

## Data Availability

All data are provided in the paper.
